# The influence of dietary supplementation with the leucine metabolite β-hydroxy-β-methylbutyrate (HMB) on the chemotaxis, phagocytosis and respiratory burst of peripheral blood granulocytes and monocytes in calves

**DOI:** 10.1186/s12917-020-02389-1

**Published:** 2020-06-01

**Authors:** Roman Wójcik, Joanna Małaczewska, Grzegorz Zwierzchowski, Jan Miciński, Edyta Kaczorek-Łukowska

**Affiliations:** 1 Department of Microbiology and Clinical Immunology, Faculty of Veterinary Medicine, University of Warmia and Mazury in Olsztyn, Oczapowskiego 13, 10-719 Olsztyn, Poland; 2 Department of Biochemistry, Faculty of Biology and Biotechnology, University of Warmia and Mazury in Olsztyn, Oczapowskiego 1A, 10-719 Olsztyn, Poland; 3 Department of Sheep and Goat Breeding, Faculty of Animal Bioengineering, University of Warmia and Mazury in Olsztyn, ul. Oczapowskiego 5, 10-917 Olsztyn, Poland

**Keywords:** β-hydroxy-β-methylbutyrate, Granulocytes, Monocytes, Chemotaxis, Phagocytosis, Respiratory burst, Calves

## Abstract

**Background:**

A healthy immune system plays a particularly important role in newborns, including in calves that are far more susceptible to infections (viral, bacterial and other) than adult individuals. Therefore, the present study aimed to evaluate the influence of HMB on the chemotactic activity (MIGRATEST® kit), phagocytic activity (PHAGOTEST® kit) and oxidative burst (BURSTTEST® kit) of monocytes and granulocytes in the peripheral blood of calves by flow cytometry.

**Results:**

An analysis of granulocyte and monocyte chemotactic activity and phagocytic activity revealed significantly higher levels of phagocytic activity in calves administered HMB than in the control group, expressed in terms of the percentage of phagocytising cells and mean fluorescence intensity (MFI). HMB also had a positive effect on the oxidative metabolism of monocytes and granulocytes stimulated with PMA (4-phorbol-12-β-myristate-13-acetate) and *Escherichia coli* bacteria, expressed as MFI values and the percentage of oxidative metabolism.

**Conclusion:**

HMB stimulates non-specific cell-mediated immunity, which is a very important consideration in newborn calves that are exposed to adverse environmental factors in the first weeks of their life. The supplementation of animal diets with HMB for both preventive and therapeutic purposes can also reduce the use of antibiotics in animal production.

## Background

Considerable research has been done into preparations that simulate immune mechanisms, in particular non-specific cell-mediated and humoral immunity, in the youngest animals [1–5]. A healthy immune system plays a particularly important role in newborns, including in calves that are far more susceptible to infections (viral, bacterial and other) than adult individuals [6–8].

Non-infectious factors, such as the season of birth, low birth weight, unfavourable environment, absence of colostrum feeding after birth or colostrum feeding at an inappropriate time, as well as infectious factors that cause gastrointestinal and respiratory diseases, are the most frequent causes of disease that contribute to high mortality in newborn calves and cause production losses [6–15]. These diseases are difficult to treat and the prognosis is doubtful, which is why prevention, including immunoprevention, could play a very important role in calf rearing [16–18]. The search for effective immunostimulants, in particular feed additives, continues, and β-hydroxy-β-methylbutyrate (HMB) could be one of such supplements.

Beta-hydroxy-β-methylbutyrate occurs naturally in humans, animals and plants. This endogenous metabolite of the branched-chain amino acid (BCAA) leucine (LEU) is produced when leucine is oxidised in the cell cytoplasm, mainly in the liver and muscles. Nearly 80% of endogenous LEU is used in protein synthesis, and the remainder is transformed into α-ketoisocaproate (α-KIC) which can undergo further conversion in two processes. The first process occurs in the mitochondria where α-KIC is oxidised to isovaleryl-CoA by branched-chain ketoacid dehydrogenase (BCKAD). 3-hydroxy-3-methylglutaryl-coenzyme A (HMG-CoA) is synthesised after several steps. An alternative process takes place in the cytosol where HMB is produced from α-KIC by the KIC dioxygenase enzyme. Under normal conditions, approximately 5% of leucine is metabolised into HMB, whereas KIC is mostly converted to isovaleryl-CoA [19, 20].

Small quantities of HMB are found in alfalfa, corn (corn silage), milk, cheese, citrus fruit (grapefruit), selected fish species (catfish), red wine and red meat. The quantity of HMB that occurs naturally in the body and is supplied with food is insufficient and, therefore, has to be supplemented from external sources [21–23]. HMB is a safe compound that does not produce side effects even if administered at excessive doses, and excess HMB is excreted with urine [24].

According to research studies conducted on several animal species: pigs [25–27], cattle [28, 29], goats [30], chickens [31], geese [32] and fish [33, 34] HMB can stimulate immune mechanisms. However, little is known about its exact mechanism of action and effects on different immunity parameters in animals, including calves. Therefore, the aim of this study was to evaluate the effect of dietary supplementation with HMB on the chemotaxis, phagocytosis and respiratory burst of monocytes and granulocytes and in the peripheral blood of calves. The present study complements our previous research into the influence of HMB on selected parameters of non-specific cell-mediated [28] and humoral immunity [29] in calves.

## Results

**Fig. 1:**
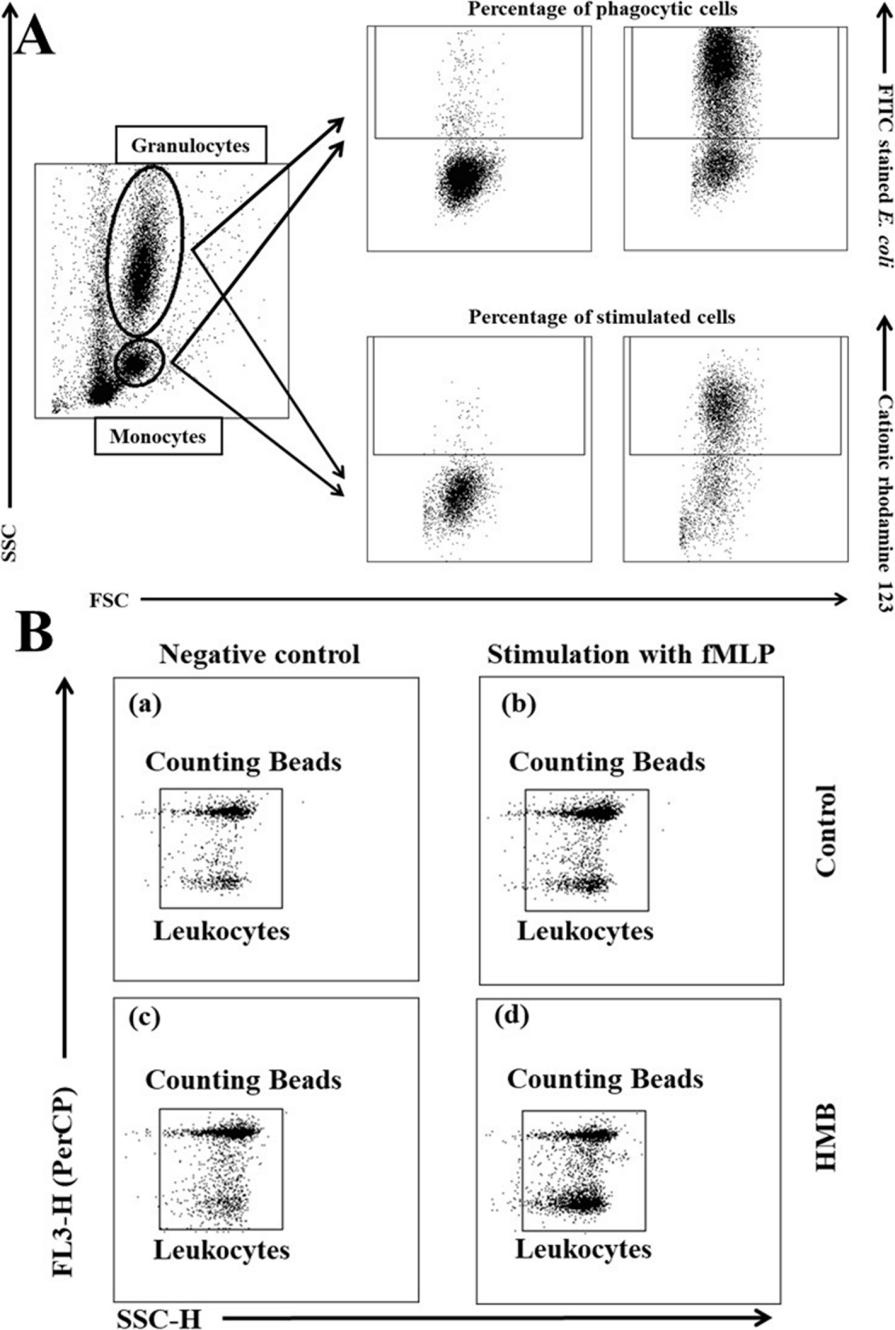
**a**. The chemotactic index was calculated by dividing the number of cells that migrated towards fMLP by the number of cells that migrated in the absence of fMLP. Key: I – control group; II – experimental group; SD - standard deviation; Numerical results were presented as the arithmetic mean ± SD. The significance level was set at 0.05. Asterisks refer to statistically significant differences between the control group and the experimental group on the same sampling day at ** *p* < 0.01; *** *p* < 0.001; C refers to statistically significant differences between day “0” and the consecutive sampling days in the experimental group at C – *p* < 0.001. **b.** Representative dot plot cytograms showing the percentage of migrating granulocytes and counting beads on day 30 of the experiment. (a) – control without stimulation; (b) – control stimulated with fMLP; (c) – HMB without stimulation; (d) – HMB stimulated with fMLP. The number of granulocytes relative to the number of whole cells was determined in each sample as soon as 2000 counting beads were acquired. Stimulation with fMLP increased the number of migrated granulocytes (b), (d) relative to the controls without fMLP (a), (c)

The chemotactic activity of peripheral blood neutrophils in calves was expressed by the chemotactic index (Fig. 1a, b). On experimental days 30 and 60, the mean values of the chemotactic index increased significantly (*p* < 0.001 and *p* < 0.01, respectively) in the group of calves whose diets were supplemented with HMB (experimental group) in comparison with the non-supplemented animals (control group). In the experimental group, the mean value of the chemotactic index also increased significantly (*p* < 0.001) on experimental days 30 and 60 relative to its mean baseline value (day 0).

**Fig. 2:**
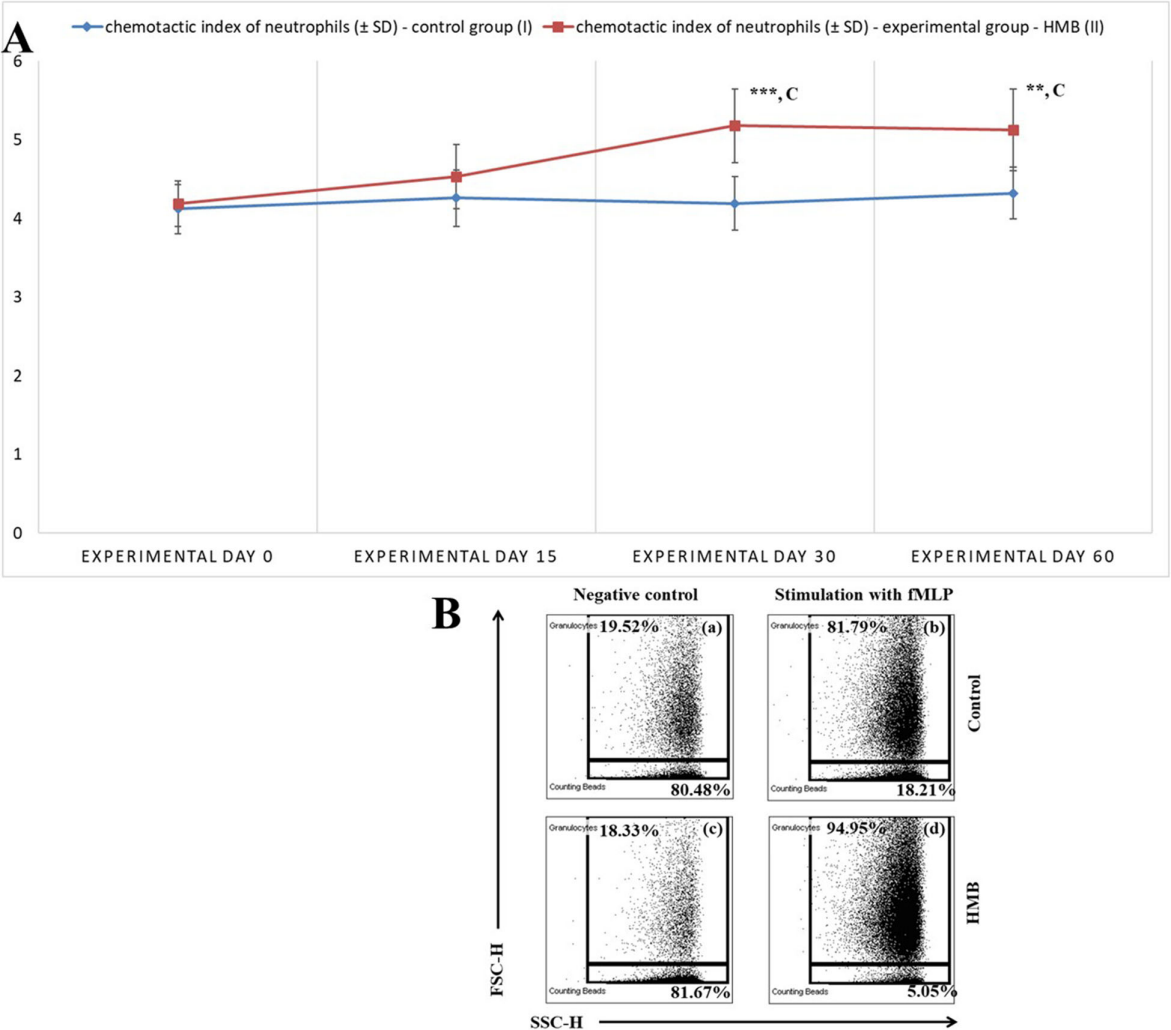
**a**. The percentage of phagocytising granulocytes in calf groups, as determined in the Phagotest® kit. Key: I – control group; II – experimental group; SD - standard deviation. Numerical results were presented as the arithmetic mean ± SD. The significance level was set at 0.05. Asterisks refer to statistically significant differences between the control group and the experimental group on the same sampling day at * *p* < 0.05; ** *p* < 0.01; A refers to statistically significant differences between day “0” and the consecutive sampling days in the experimental group at A – *p* < 0.05. **b.** Dot plot cytogram showing the percentage of phagocytising granulocytes in control and experimental calves on experimental day 30. Whole heparinised blood from control and experimental animals was incubated for 10 min with FITC-labelled *E. coli* in an ice bath at a temperature of 0 °C (negative control) or in a water bath at a temperature of 37 °C (control and HMB). The percentages of granulocytes with ingested *E. coli* (FITC) bacteria were gated. **c.** Mean fluorescence intensity (MFI) of granulocytes in calf groups, as determined in the Phagotest® kit. Key: I – control group; II – experimental group; SD - standard deviation; Numerical results were presented as the arithmetic mean ± SD. The significance level was set at 0.05. Asterisks refer to statistically significant differences between the control group and the experimental group on the same sampling day at *** *p* < 0.001; B refers to statistically significant differences between day “0” and the consecutive sampling days in the experimental group at B – *p* < 0.01

The analysis of the phagocytic activity of peripheral blood neutrophils revealed that throughout the study, the mean percentage of phagocytising neutrophils was significantly higher (*p* < 0.05 or *p* < 0.01) (Fig. 2a, b) in experimental calves than in control animals. In the experimental group, the mean percentage of phagocytising neutrophils increased significantly (*p* < 0.05) on days 30 and 60 relative to the mean baseline value of this parameter on day 0. The MFI of granulocytes, which denotes the number of ingested bacteria per phagocyte (Fig. 2C), increased significantly (*p* < 0.001, *p* < 0.01 respectively) between experimental days 15 and 30 in the experimental group relative to the control group and relative to the mean baseline value (day 0).

**Fig. 3:**
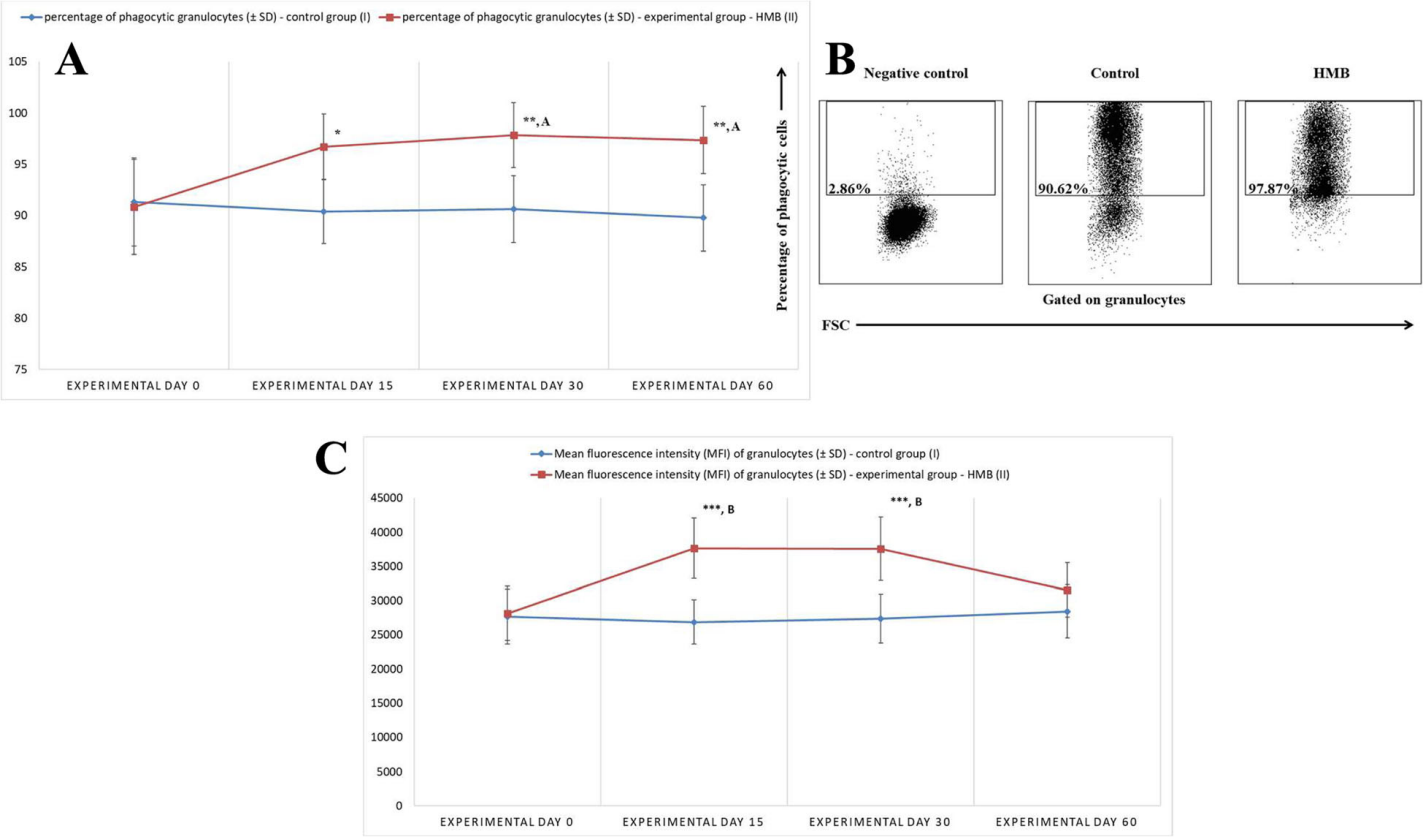
**a**. The percentage of phagocytising monocytes in calf groups, as determined in the Phagotest® kit. Key: I – control group; II – experimental group; SD - standard deviation. Numerical results were presented as the arithmetic mean ± SD. The significance level was set at 0.05. Asterisks refer to statistically significant differences between the control group and the experimental group on the same sampling day at *** *p* < 0.001; **** *p* < 0.0001; D refers to statistically significant differences between day “0” and the consecutive sampling days in the experimental group at D – *p* < 0.0001. **b.** Dot plot cytogram showing the percentage of phagocytising monocytes in control and experimental calves on experimental day 30. Whole heparinised blood from control and experimental animals was incubated for 10 min with FITC-labelled *E. coli* in an ice bath at a temperature of 0 °C (negative control) or in a water bath at a temperature of 37 °C (control and HMB). The percentages of monocytes with ingested *E. coli* (FITC) bacteria were gated. **c.** Mean fluorescence intensity (MFI) of monocytes in calf groups, as determined in the Phagotest® kit. Key: I – control group; II – experimental group; SD - standard deviation. Numerical results were presented as the arithmetic mean ± SD. The significance level was set at 0.05. Asterisks refer to statistically significant differences between the control group and the experimental group on the same sampling day at *** *p* < 0.001; **** *p* < 0.0001; D refers to statistically significant differences between day “0” and the consecutive sampling days in the experimental group at D – *p* < 0.0001

The phagocytic activity of peripheral blood monocytes, expressed as the mean percentage of phagocytising monocytes (Fig. 3a, b), on experimental days 30 and 60 increased significantly (*p* < 0.0001, *p* < 0.001 respectively) in the experimental group relative to the control group and relative to the mean baseline value (day 0). Similarly to neutrophils, the MFI of monocytes, which denotes the number of ingested bacteria per phagocyte (Fig. 3c), increased significantly (*p* < 0.0001, *p* < 0.001 respectively) between experimental days 15 and 30 in the experimental group relative to the control group and relative to the mean baseline value (day 0).

**Fig. 4:**
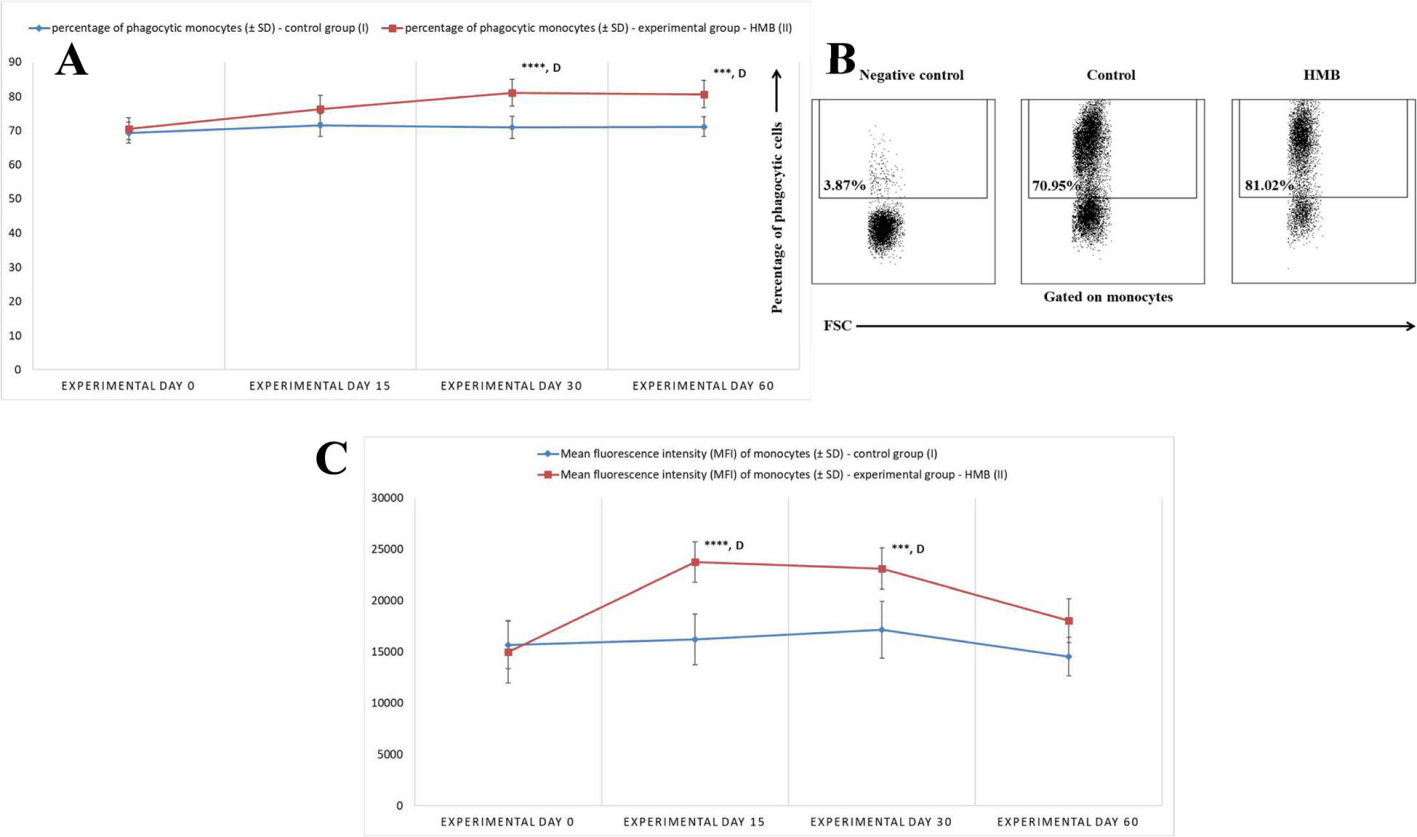
**a** The percentage of granulocytes stimulated to undergo respiratory burst in calf groups after stimulation with fMLP, PMA and *E. coli*, as determined in the Bursttest® kit. Key: I – control group; II – experimental group; SD - standard deviation. Numerical results were presented as the arithmetic mean ± SD. The significance level was set at 0.05. Asterisks refer to statistically significant differences between the control group and the experimental group on the same sampling day at * *p* < 0.05; ** *p* < 0.01; A, B refer to statistically significant differences between day “0” and the consecutive sampling days in the experimental group at A – *p* < 0.05; B – *p* < 0.01. **b.** Dot plot cytogram showing the percentage of granulocytes stimulated to undergo respiratory burst in control and experimental calves on experimental day 30. Whole heparinised blood from control and experimental animals (control and HMB) was divided into four test tubes. The samples were combined with the washing solution (negative control), *E. coli* bacteria (opsonising activator), PMA (strong activator) or fMLP (weak activator) and incubated with dihydrorhodamine 123 in a water bath at a temperature of 37 °C. After incubation, cells were lysed and DNA staining solution was added. The percentages of granulocytes stimulated to undergo respiratory burst (conversion of dihydrorhodamine 123 to rhodamine 123) were gated. **c.** Mean fluorescence intensity (MFI) of granulocytes in calf groups after stimulation with fMLP, PMA and *E. coli*, as determined in the Bursttest® kit. Key: I – control group; II – experimental group; SD - standard deviation. Numerical results were presented as the arithmetic mean ± SD. The significance level was set at 0.05. Asterisks refer to statistically significant differences between the control group and the experimental group on the same sampling day at * *p* < 0.05; ** *p* < 0.01; *** *p* < 0.001; **** *p* < 0.0001; B, C, D refer to statistically significant differences between day “0” and the consecutive sampling days in the experimental group at B – *p* < 0.01; C – *p* < 0.001; D – *p* < 0.0001

The respiratory burst activity (metabolism of highly reactive oxygen species) of peripheral blood neutrophils increased in the experimental group fed HMB-supplemented diets relative to the control group and relative to the mean baseline value (day 0) throughout the experiment. An increase was observed in the mean percentage of cells stimulated to undergo respiratory burst (Fig. 4a, b) and in the MFI of neutrophils denoting the respiratory burst activity of different neutrophils (Fig. 4c). However, a significant (*p* < 0.05 or *p* < 0.01 or *p* < 0.001 or *p* < 0.0001) increase in the mean values of the above parameters was observed only after stimulation with potent respiratory burst activators, PMA and *E. coli* bacteria, throughout the experiment. The stimulation with N-formyl-methionyl-leucyl-phenylalanine (fMLP), a weak activator of respiratory burst, did not induce significant differences in the mean percentage of cells stimulated to undergo respiratory burst or the MFI of neutrophils between the control group and the experimental group or relative to the mean baseline values (day 0) (Fig. 4a).

**Fig. 5:**
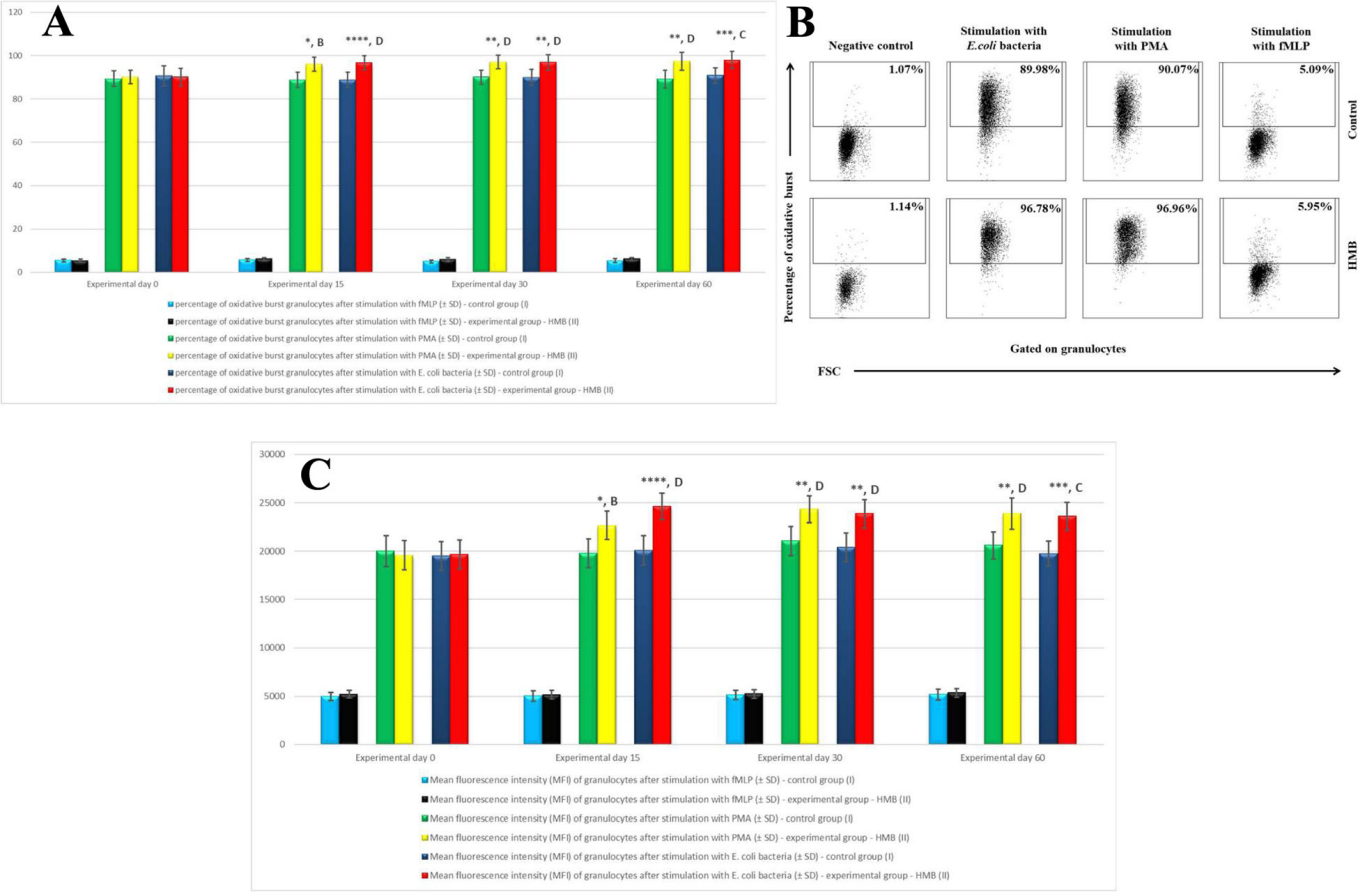
**a** The percentage of monocytes stimulated to undergo respiratory burst in calf groups after stimulation with fMLP, PMA and *E. coli*, as determined in the Bursttest® kit. Key: I – control group; II – experimental group; SD - standard deviation. Numerical results were presented as the arithmetic mean ± SD. The significance level was set at 0.05. Asterisks refer to statistically significant differences between the control group and the experimental group on the same sampling day at * *p* < 0.05; ** *p* < 0.01; *** *p* < 0.001; B, C refer to statistically significant differences between day “0” and the consecutive sampling days in the experimental group at B – *p* < 0.01; C – *p* < 0.001. **b**. Dot plot cytogram showing the percentage of monocytes stimulated to undergo respiratory burst in control and experimental animals on experimental day 30. The percentages of monocytes stimulated to undergo respiratory burst were gated. **c.** Mean fluorescence intensity (MFI) of monocytes in calf groups after stimulation with fMLP, PMA and *E. coli*, as determined in the Bursttest® kit. Key: I – control group; II – experimental group; SD - standard deviation; Numerical results were presented as the arithmetic mean ± SD. The significance level was set at 0.05. Asterisks refer to statistically significant differences between the control group and the experimental group on the same sampling day at * *p* < 0.05; ** *p* < 0.01; A, B, C refer to statistically significant differences between day “0” and the consecutive sampling days in the experimental group at A – *p* < 0.05; B – *p* < 0.01; C – *p* < 0.001

Similarly to granulocytes, the stimulation of peripheral blood monocytes with *E. coli*, PMA and fMLP significantly (*p* < 0.05 or *p* < 0.01 or *p* < 0.001) increased the mean percentage of cells stimulated to undergo respiratory burst (Fig. 5a, b) and the mean intensity of respiratory burst in monocytes (Fig. 5c) in the experimental group relative to the control group and relative to the mean baseline values (day 0).

## Discussion

This study evaluated the effect of the supplementation of calf diets with HMB, a natural metabolite of leucine, at different stages of phagocytosis: chemotaxis and intracellular ingestion and killing of bacteria by peripheral blood granulocytes and monocytes.

There is considerable evidence to indicate that HMB stimulates non-specific immune mechanisms, including phagocytosis, in many animal species: pigs [35], chicken [31, 36], geese [32] and fish [34, 37, 38] however, these effects have been rarely investigated in calves [28]. HMB is a strong anticatabolic agent and a regulator of protein metabolism, and it is widely used in sports and bodybuilding to increase strength, muscle mass and exercise performance [39–43]. Immune health is also highly correlated with protein synthesis because mounting immune responses require the generation of new cells and the synthesis of antigen-presenting machinery, immunoglobulins, cytokines, cytokine receptors and acute phase proteins.

In the present study, the observed increase in the chemotactic activity of peripheral blood neutrophils, expressed as the chemotactic index as the most accurate parameter for assessing chemotactic activity [44], in the group of experimental calves supplemented with HMB relative to control group calves cannot be compared with published data because such experiments have not been conducted in calves or in other animal species. As mentioned previously, numerous authors [28, 31, 32, 34–38] have reported on the positive effects of HMB on phagocytosis in several animal species, but the supplement’s impact on specific stages of phagocytosis has never been described. It should be noted that unlike in humans, typical fMLP receptors are not expressed on the surface of WBC in cattle, and this fact has not been disputed in this study. However, certain doubts remain, and they appear to be backed by the literature. The theory that typical fMLP receptors are not expressed on the surface of bovine WBC was postulated in the last century, and the research conducted in the past decade has provided evidence to the contrary. These findings could be insignificant, but in our opinion, they are worth mentioning. The responses of bovine granulocytes to the fMLP chemoattractant in the MIGRATEST® suggest that these cells are bound to a receptor. However, this receptor has not been identified, and it appears that other receptors present on the surface of bovine WBC could play the role of typical fMLP receptors or that co-receptors could be involved. These are merely speculations because such receptors have not yet been described.

The significant increase in the phagocytic activity of monocytes and granulocytes in the experimental group relative to the control group was reflected by an increase in the percentage of phagocytising cells as well as an increase in the mean number of ingested bacteria per phagocyte, expressed as MFI. Similar results were noted in our previous studies of geese [32] and goats [45]. In a study by Siwicki et al. [37], the potential killing activity (PKA) of granulocytes and monocytes increased by 140% in fish (rainbow trout and carp) stimulated with various doses of HMB relative to the control group. The analysed supplement exerted the most stimulatory effect on PKA when administered at doses higher than 50 mg HMB/mL. A study of rainbow trout [38] fed pellets with various HMB content (0, 10, 25 or 50 mg/kg BW/day) for 8 weeks confirmed that HMB stimulated the activity of phagocytes (100% increase in PKA), in comparison with the control treatment. Similar observations were made by Siwicki et al. [46] who examined the effect of two HMB doses (50 and 100 mg HMB/kg BW/day) administered over a period of 4 weeks on non-specific cell-mediated immunity in the common carp (*Cyprinus carpio*). The PKA of pronephric phagocytes was significantly higher (*p* < 0.05) in HMB-fed carp than in the control group. However, significant differences were not observed between experimental carp administered HMB doses of 50 and 100 mg/kg BW/day.

In the current study, HMB also significantly enhanced the intracellular killing activity of granulocytes and monocytes stimulated with strong mitogens (PMA and *E. coli* bacteria), which was expressed by an increase in the percentage of cells stimulated to undergo respiratory burst and in MFI in these cells. Higher values of the above parameters indicate that pathogens were more effectively eliminated from the body by phagocytising cells. Similar results were reported by Siwicki et al. [38], where a spectrophotometric analysis in the respiratory burst activity test revealed that the production of highly reactive oxygen species by head kidney phagocytes doubled in rainbow trout whose diets were supplemented with HMB for 8 weeks. In a study of rainbow trout (*Oncorhynchus mykiss*) and carp (*Cyprinus carpio*) [40], the addition of > 50 mg/ml HMB to the culture medium increased respiratory burst activity by up to 84% (*p* < 0.01) in pronephric phagocytes cultured in the RPMI-1640 medium supplemented with 0, 0.1, 1, 5, 10, 25, 50 or 100 mg HMB/mL relative to the cells cultured without HMB. In an in vitro study, Peterson et al. [36] evaluated macrophages that were isolated from a chicken macrophage cell line (MQ-NCSU) and cultured in the presence of 20, 40 and 80 μg of HMB. Supernatant fractions were also tested for the presence of nitrite. The phagocytic potential of MQ-NCSU macrophages exposed to 40 μg of HMB was significantly higher (31.7%) relative to the controls. Sephadex-elicited macrophages treated with 80 μg HMB exhibited a 14.4% increase in phagocytosis compared with the controls.

The mechanism by which HMB influences phagocytising cells and other immunocompetent cells, isolated in this experiment, has not yet been fully elucidated. It can be hypothesised that as a leucine catabolite, HMB is an abundant source of energy for biological activities [19] or that it is an effector of energy metabolism.

The results of this study suggest that HMB is a highly effective dietary supplement that stimulates the immune system of animals. HMB stimulates non-specific cell-mediated immunity, which is a very important consideration in newborn calves that are exposed to adverse environmental factors in the first weeks of their life. The supplementation of animal diets with HMB for both preventive and therapeutic purposes can also reduce the use of antibiotics in animal production.

Although we are aware that the number of animals enrolled in the present study could be considered a limitation, we would like to emphasise that the study was not performed in a large research centre, but in an academic unit with a limited budget; therefore, all experimental materials had to be managed rationally. Cytometry tests were a considerable financial burden, even with such a small number of animals. We hope to conduct similar research in the future on a larger number of animals and with a higher number of evaluated parameters because the discussed topic seems to be highly interesting.

## Conclusions

The results of this study indicate that experimental calves fed diets supplemented with HMB, compared with non-supplemented (control) animals, were characterized by significantly higher levels of granulocyte chemotactic activity (cf. the chemotactic index) and phagocytic activity (cf. the percentage of phagocytes), a significantly higher mean number of ingested bacteria per phagocyte (cf. the mean fluorescence intensity of monocytes and granulocytes), and significantly higher levels of intracellular killing activity of monocytes and granulocytes after stimulation with PMA and *E. coli* (cf. the percentage of stimulated cells and mean fluorescence intensity).

## Methods

### Experimental design

The experiment was performed on 14 Polish Holstein-Friesian calves originating from a private dairy herd in north-eastern Poland. We are aware that the studied population was rather small; however, the evaluated animals are large, and the number of animals per group fully meets the criteria for conducting research of the type. The calves (aged 30 ± 2 days) were fed colostrum within 1 h after birth, at 2 kg/animal/day for 5 days. Next, the animals were fed milk replacer until 8 weeks of age. Immediately after birth, the calves were moved to wooden sheds outside the cow barn. One-month-old calves were included in a 60-day study. Prior to the experiment, the animals were weighed and randomly allocated to two groups by the analogue method. The control group (I) comprised calves fed a standard farm-made diet. Between 5 days and 8 weeks of age, they were fed the Mlekowit (Polmass, Bydgoszcz, Poland) milk replacer at 4 L/day/animal in two portions. In the experimental group (II), the same quantity of the milk replacer was supplemented with β-hydroxy-β-methylbutyrate (HMB, Metabolic Technologies Inc. Ames, IA, USA) at 40 mg/kg BW. Beginning from the first week, the animals had unlimited access to solid feed (maize silage, meadow hay and Jösera Kälberkost prestarer) which was supplied in increasing quantities. Fresh water was provided ad libitum. All calves enrolled in this study survived the experimental period and remained in the herd.

### Sample collection

Blood was sampled from the jugular vein prior to HMB supplementation and on days 15, 30 and 60 of the experiment to determine and compare the chemotactic activity (MIGRATEST® kit) (Glycotope Biotechnology GmbH, Heidelberg, Germany), phagocytic activity (PHAGOTEST® kit) (Orpegen Pharma, Heidelberg, Germany) and respiratory burst (BURSTTEST® kit) (Orpegen Pharma, Heidelberg, Germany) of monocytes and granulocytes in the peripheral blood of calves by flow cytometry. The methods used for analyzing white blood cell functions are only briefly summarized in the present paper. For a more detailed description, please refer to Wójcik et al. [45].

### Determination of blood monocyte and granulocyte chemotaxis in calves with the MIGRATEST® kit

The chemotactic activity of blood granulocytes and monocytes was determined with the MIGRATEST® kit (Orpegen Pharma, Heidelberg, Germany) based on the attached instructions. The assay involved leucocyte-rich plasma (LRP) that was isolated from heparinised whole blood by spontaneous sedimentation. The LRP was extracted from each sample, and it was transferred to two cell culture inserts (pore size: 3.0 μM). One of the inserts was placed in a well containing N-formyl-methionyl-leucyl-phenylalanine (fMLP) (Glycotope Biotechnology GmbH, Heidelberg, Germany) as the chemoattractant. The other insert was used as negative control, and it was placed in a buffer solution that did not contain the chemotactic peptide. Chemotaxis was quantified over a period of 30 min at a temperature of 37 °C. An antibody reagent (FITC-labelled anti-CD62L (Glycotope Biotechnology GmbH, Heidelberg, Germany) containing counting beads was applied for 10 min to stain the cells. The samples were placed on ice, and vital DNA dye (Glycotope Biotechnology GmbH, Heidelberg, Germany) was added for 5 min before flow cytometry. The number of migrated neutrophils and L-selectin shedding from the surface of activated cells were determined by flow cytometry. The results were expressed in terms of the chemotactic index, defined as the number of cells that migrated towards fMLP divided by the number of cells that migrated in the absence of fMLP [47].

### Determination of blood monocyte and granulocyte phagocytosis in calves with the PHAGOTEST® kit

The phagocytic activity of blood granulocytes and monocytes was evaluated with the use of the PHAGOTEST® kit (Orpegen Pharma, Heidelberg, Germany) in line with the attached instructions. The manufacturer’s specifications were also observed in the process of preparing the reagents. The negative control was a 5 mL test tube (blue, Beckman Coulter, Fullerton, CA, USA) filled with 100 μL of whole heparinised blood chilled to 0 °C. The experimental test tube (5 mL, blue, Beckman Coulter, Fullerton, CA, USA) contained 20 μL of cooled *E. coli* bacteria (Orpegen Pharma, Heidelberg, Germany). Both tubes were shaken at low speed for approximately 3 s. The experimental test tube was incubated at 37 °C for 10 min. The negative control was incubated at 0 °C on ice. The incubated samples were combined with 100 μL of the quenching solution (Orpegen Pharma, Heidelberg, Germany), and the test tubes were shaken. The washing solution (Orpegen Pharma, Heidelberg, Germany) chilled to 0 °C was added in the amount of 3 ml. Both samples were centrifuged for 5 min at 4 °C (250 x g), and the supernatant was removed. The samples were rinsed twice, and 2 ml of the lysing solution (Orpegen Pharma, Heidelberg, Germany) with room temperature was added to each sample. The samples were shaken, incubated at room temperature for 20 min, and centrifuged for 5 min at 4 °C (250 x g). The supernatant was removed. Each sample was combined with 3 mL of the washing solution (Orpegen Pharma, Heidelberg, Germany) with a temperature of 0 °C. After centrifugation for 5 min at 4 °C (250 x g), the supernatant was discarded. A DNA staining solution (Orpegen Pharma, Heidelberg, Germany) chilled to 0 °C was added in the amount of 200 μL. The samples were shaken and incubated on ice for 10 min. The phagocytic activity of the evaluated cells was measured in a cytometer (Beckmann Coulter, Epics XL, USA) within less than 60 min after the addition of the last reagent. The Phagotest kit (Orpegen Pharma, Heidelberg, Germany) contains fluorescein (FITC)-stained and phagocytised *E. coli* bacteria. The cell nuclei were also stained. The number of phagocytising cells, granulocytes and monocytes are determined separately. Phagocytic activity is evaluated based on the mean fluorescence intensity (MFI) of individual cells that ingest bacteria.

### Determination of the oxidative metabolism of blood monocytes and granulocytes in calves with the BURSTTEST® kit

The oxidative metabolism of blood granulocytes and monocytes was determined with the BURSTTEST (Orpegen Pharma, Heidelberg, Germany) based on the attached instructions. The manufacturer’s specifications were also observed in the process of preparing the reagents. Whole heparinised blood was split into four portions and placed in four 100 μL test tubes (blue, Beckman Coulter, Fullerton, CA, USA) chilled to 0 °C. The first sample (experimental) was combined with 20 μL of chilled *E. coli* bacteria (Orpegen Pharma, Heidelberg, Germany); the second sample (negative control) was combined with 20 μL of the washing solution (Orpegen Pharma, Heidelberg, Germany); the third sample (low control) was combined with 20 μL of fMLP (N-formyl-methionyl-leucyl-phenylalanine) (Orpegen Pharma, Heidelberg, Germany); the fourth sample (high control) was combined with 20 μL of PMA (4-phorbol-12-β-myristate-13-acetate) (Orpegen Pharma, Heidelberg, Germany). The contents of each test tube were stirred and incubated at 37 °C for 10 min, excluding the sample containing fMLP which was incubated for 7 min. Twenty μL of substrate solution (Orpegen Pharma, Heidelberg, Germany) was added to each incubated sample, and the tubes were thoroughly shaken. The samples were incubated at 37 °C for 10 min, and 2 mL of the lysing solution (Orpegen Pharma, Heidelberg, Germany) at room temperature was added to each test tube. The samples were shaken and incubated for 20 min. at room temperature. After centrifugation for 5 min at 4 °C (250×g), the supernatant was discarded. The samples were rinsed once with 3 ml of the washing solution (Orpegen Pharma, Heidelberg, Germany), they were centrifuged at 4 °C for 5 min (250×g), and the supernatant was removed. Each sample was combined with 200 μL of the staining solution chilled to 0 °C, it was shaken and incubated for 10 min on ice. The intracellular killing activity of phagocytes was measured by flow cytometry (BD Biosciences, San Jose, California, USA) within less than 30 min after the addition of the last reagent. The cells were stimulated with three activators: *E. coli* bacteria (Orpegen Pharma, Heidelberg, Germany), PMA (Orpegen Pharma, Heidelberg, Germany) as the strong activator, and fMLP (Orpegen Pharma, Heidelberg, Germany) as the weak activator. Oxidative stress was induced with H_2_O_2_, and mitochondria-generated reactive oxygen species converted dihydrorodamine (123-DHR) to rhodamine 123 (R123), a florescent cationic dye.

### FACS acquisition and analysis

**Fig. 6:**
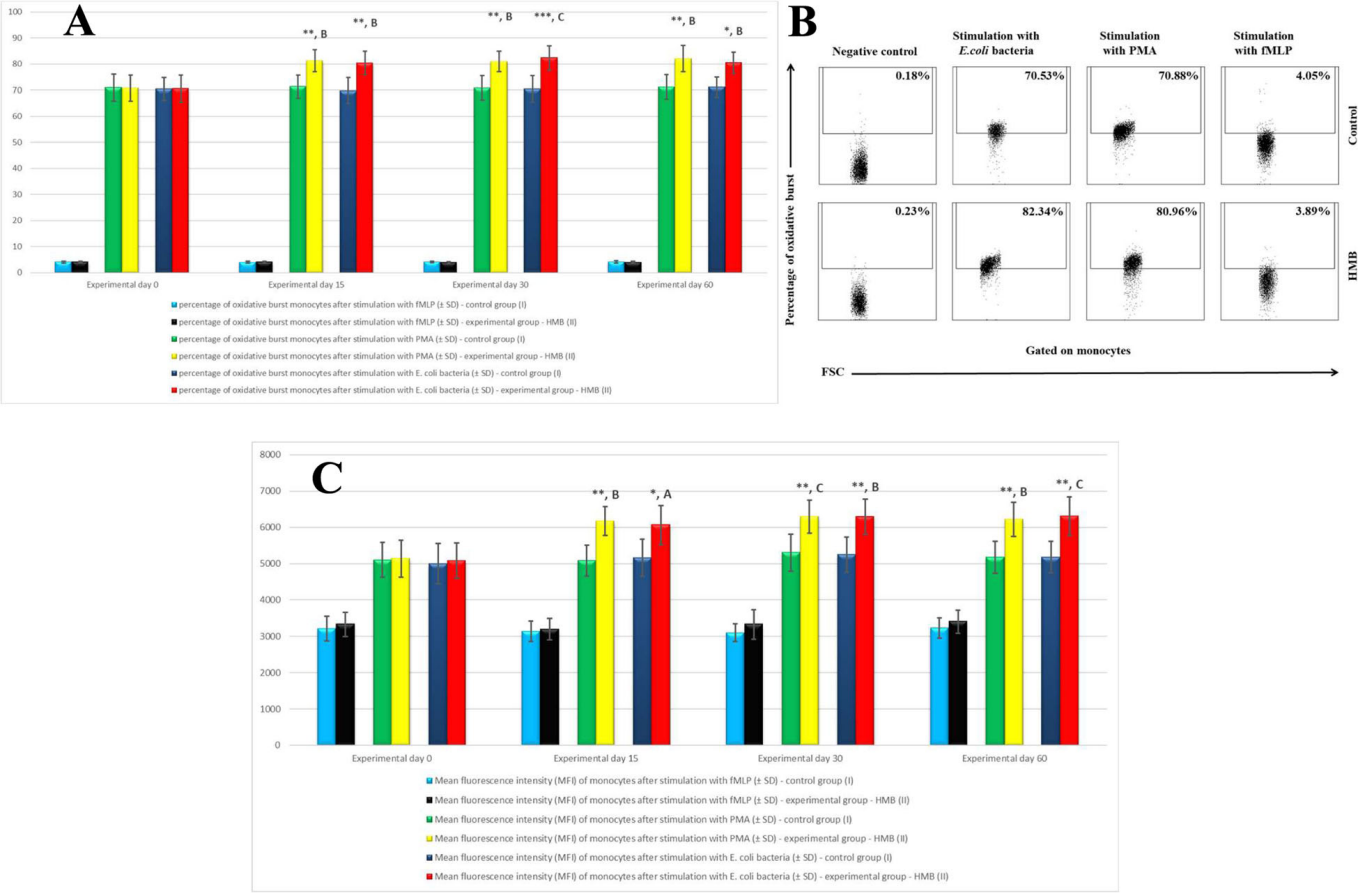
**a**. Gating strategy for flow cytometry data analysis. Granulocytes and monocytes were gated based on forward and side scatter (FSC/SSC) parameters. Each cell subset was analysed for the relative number of phagocytising cells and cells stimulated for respiratory burst (fMLP, PMA or *E. coli* bacteria). **b.** Gating strategy for analysing flow cytometry data based on the neutrophil migration assay. Granulocytes and counting beads were identified in PerCP to SSC scatter and depicted in FSC to SSC scatter. (a) – control without stimulation; (b) – control stimulated with fMLP; (c) – HMB without stimulation; (d) – HMB stimulated with fMLP

A flow cytometric analysis was conducted using the FACSCanto II cytometer (BD Biosciences, San Jose, California, USA). Data were acquired using FACSDiva version 6.1.3 software (BD Biosciences, San Jose, California, USA), and they were analysed using FlowJo 10 software (Tree Star, Ashland, Oregon, USA). Photomultiplier tube (PMT) settings were adjusted using the cytometry setup and tracking beads (CST; BD Biosciences, San Jose, California, USA). Flow cytometric compensation was established using a single stain control for each fluorochrome and unstained (control) cells. Monocytes and granulocytes were gated based on their forward and side scatter (FSC/SSC) parameters (Fig. 6a).

Granulocytes and counting beads were detected on PerCP to SSC scatter, and depicted in FSC to SSC scatter. Data acquisition was completed when exactly 2000 events were acquired in the region of counting beads. The number of events in the region of granulocytes was counted, and the number of granulocytes in the control sample was compared with the number of granulocytes in the positive control sample after stimulation with fMLP. The decrease in L-selectin expression could be measured simultaneously. Down-regulation of this cell adhesion molecule is directly correlated with the activation of neutrophils in response to chemotactic factors. Cell migration is preceded by changes in cell shape, which can be determined based on the changes in forward scatter signals observed during flow cytometry (Fig. 6b).

### Statistical analysis

Numerical data were presented as the arithmetic mean ± SD. The results were analysed statistically by two-way ANOVA for orthogonal designs. Post-hoc tests were performed using GraphPad Prism 7 software. Day 0 was compared with days 15, 30, and 60 in group II by Dunnett’s test (significant inter-day differences: (A) *p* < 0.05; (B) *p* < 0.01; (C) *p* < 0.001; (D) *p* < 0.0001). Group II was compared with group I at each time point by Tukey’s test for equal groups (significant inter-group differences: * *p* < 0.05; ** *p* < 0.01; *** *p* < 0.001; **** *p* < 0.0001). The significance level was set at 5% HMB.

## Data Availability

The datasets used and/or analysed during the current study are available from the corresponding author on reasonable request.

## Abbreviations

BCAA: Branched-chain amino acid
BW: Body weight
CD: Cluster of differentiation
CR3: Complement receptor 3
fMLP: N-formyl-methionyl-leucyl-phenylalanine
GC-SF: Granulocyte colony-stimulating factor
HMB: β-hydroxy-β-methylbutyrate
HMB-FA: β-hydroxy-β-methylbutyrate free acid
HMG-CoA: 3-hydroxy-3-methylglutaryl-coenzyme A
IFN-γ: Interferon-γ
IL: Interleukin
LEU: Leucine
MFI: Mean fluorescence intensity
PKA: Potential killing activity
PMA: 4-phorbol-12-β-myristate-13-acetate
SR-A: Class A scavenger receptors
TNFR1: TNF-α receptor 1
TNF-α: Tumour necrosis factor α
α-KIC: α-ketoisocaproate
